# Triglyceride-glucose index is associated with the risk of impaired fasting glucose in Chinese elderly individuals

**DOI:** 10.1038/s41598-024-67081-y

**Published:** 2024-07-11

**Authors:** Jie Liu, Feng Yi, Kai Duan, Haibo Liu

**Affiliations:** 1 Department of Emergency Medicine, Shenzhen New Frontier United Family Hospital, Shenzhen, 518000 China; 2 Department of Emergency Medicine, Yueyang Central Hospital, Yueyang, 414000 Hunan China

**Keywords:** TyG index, Impaired fasting glucose, Retrospective cohort study, Chinese elder adults, Endocrinology, Risk factors

## Abstract

The association between the triglyceride-glucose (TyG) index and impaired fasting glucose (IFG) in elderly individuals remains uncertain. Our study aimed to explore the association between the TyG index and the risk of future IFG in this population. This retrospective cohort study included 17,746 elderly individuals over 60. In this population, Cox regression models proportional to hazards, along with smooth curve fitting and cubic spline functions, were employed to examine the association between the baseline TyG index and the risk of IFG. Subgroup analyses and sensitivity were also performed to ensure the robustness of the study findings. After adjusting for covariates, a positive association between the TyG index and the risk of IFG was found (HR = 1.43, 95% CI 1.27–1.60, P < 0.0001). The likelihood of IFG rose steadily as the TyG index quartiles (from Q1 to Q4) increased, with Q4 demonstrating a 62% elevated risk compared to Q1 (adjusted HR = 1.62, 95% CI 1.37–1.90). Additionally, we found the association between TyG index and risk of IFG was a linear. Sensitivity and subgroup analyses confirmed the stability of the results. Our study observed a linear association between the TyG index and the development of IFG in elderly Chinese individuals. Recognizing this association can help clinicians identify high-risk individuals and implement targeted interventions to reduce their risk of progressing to diabetes.

## Introduction

Impaired fasting glucose (IFG) is a glycemic state between normality and diabetes, defined by the American Diabetes Association (ADA) as fasting plasma glucose levels ranging from 5.6 to 6.9 mmol/L^1^. IFG is prevalent in the elderly globally, more so than diabetes itself in this demographic^2,3^. IFG not only increases the risk of developing type 2 diabetes but is also closely associated with elevated cardiovascular disease risk^4–7^. Additionally, research suggests a potential link between IFG and increased all-cause mortality^8^. Therefore, early identification and intervention in IFG risk factors are crucial for reducing disease burden.

Metabolic abnormalities, such as hyperuricemia and dyslipidemia, are closely associated with IFG. Uric acid has been identified as a component of metabolic syndrome^9^ and demonstrates a strong predictive value for hypertension, proteinuria, and peripheral artery disease^10–12^. Furthermore, uric acid is highly correlated with insulin levels, suggesting that it may serve as a potential biomarker for insulin resistance^13^. High triglycerides (TG) are an important component of dyslipidemia, closely associated with non-alcoholic fatty liver disease, insulin resistance, and atherosclerosis^14–16^. Studies have shown that any type of dyslipidemia is associated with an increased risk of developing diabetes^17^. High TG may impair glucose metabolism in muscles, reduce insulin sensitivity and metabolic status, and exacerbate insulin resistance^18^. Therefore, maintaining appropriate triglyceride levels is crucial for preventing insulin resistance, glucose metabolic disorders, and related cardiovascular diseases.

Recently, a blood glucose metabolism index, the triglyceride-glucose (TyG) index, calculated as the product of TG levels and fasting plasma glucose (FPG)^19^, has emerged as a convenient measure for assessing an individual’s degree of insulin resistance^20^. The TyG index is closely associated with various diseases. Studies have shown that the TyG index is positively correlated with the incidence of cardiovascular diseases such as carotid atherosclerosis, coronary artery disease, hypertension, and myocardial infarction^21^. Research has emphasized that the TyG index is one of the most effective markers for diagnosing metabolic syndrome^22^. Some studies have indicated that the TyG index is closely related to the occurrence and prognosis of diabetes^23,24^. Overall, the TyG index has important clinical significance in predicting cardiovascular diseases, glucose metabolic disorders, and other related diseases.

However, there is insufficient evidence regarding the association between the TyG index and risk of IFG among the elderly. Our study aims to investigate this association in individuals aged 60 and above, endorsing the TyG index’s clinical utility for early IFG detection, potentially influencing diabetes management and preventive healthcare in the elderly.

## Results

### Baseline characteristics of participants

**Figure 1:**
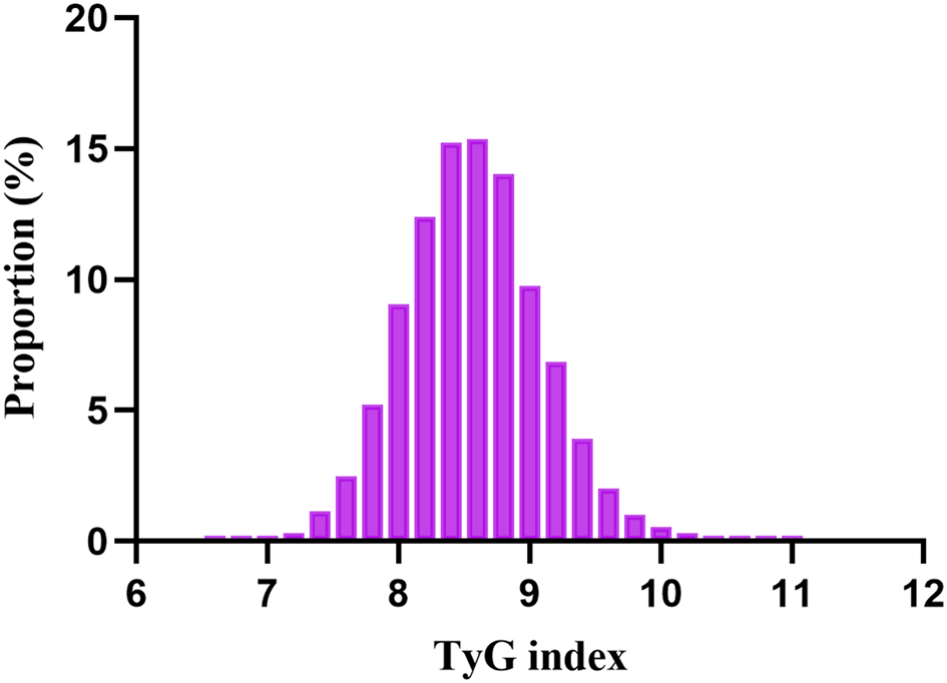
Distribution of TyG index. It presented a normal distribution, ranging from 6.61 to 11.05, with a mean of 8.57.

The baseline characteristics of the study participants were summarized as showed in Table 1. The mean age was 67.01 ± 6.49 years, with 9751 participants (54.94%) being male. The mean follow-up time was 3.13 years, and 4063 individuals (22.89%) ultimately developed IFG. The study participants were grouped into IFG and Normal groups, with the IFG group displaying significantly higher mean age and BMI compared to the Normal group (P < 0.001). This group also had elevated SBP and DBP, as well as higher FBG levels (P < 0.001). Further differences included higher TG levels, lower HDL-c levels, and lower LDL cholesterol in the IFG group (P < 0.001). Additionally, ALT and Scr levels were significantly higher in the IFG group (P < 0.001). Sex distribution and smoking status also differed significantly between the two groups, while no significant differences were observed in drinking status and family history of diabetes. Notably, the follow-up duration was longer in the IFG group (P < 0.001). The distribution of the TyG index, as shown in Fig. 1, indicated a normal distribution ranging from 6.61 to 11.05, with a mean of 8.57.

**Table 1:** The baseline characteristics of participants.

Group	Normal	IFG	P-value
Participants	13,683	4063	
Age (years)	66.87 ± 6.45	67.47 ± 6.61	< 0.001
BMI (kg/m^2^)	23.77 ± 2.97	24.63 ± 3.06	< 0.001
SBP (mmHg)	130.10 ± 18.64	134.83 ± 18.95	< 0.001
DBP (mmHg)	77.97 ± 11.04	79.95 ± 11.55	< 0.001
FBG at baseline (mmol/L)	4.84 ± 0.46	5.05 ± 0.40	< 0.001
TG (mmol/L)	1.53 ± 0.92	1.64 ± 0.98	< 0.001
TyG index	8.54 ± 0.52	8.65 ± 0.53	< 0.001
ALT (U/L)	21.02 ± 13.62	22.99 ± 15.85	< 0.001
AST (U/L)	25.65 ± 9.70	26.37 ± 11.66	0.008
BUN (mmol/L)	5.17 ± 1.27	5.17 ± 1.25	0.852
Scr (μmol/L)	72.56 ± 20.05	74.49 ± 16.59	< 0.001
TC (mmol/L)	5.16 ± 0.94	5.16 ± 0.95	0.753
HDL-c (mmol/L)	1.39 ± 0.32	1.37 ± 0.30	0.002
LDL-c (mmol/L)	3.03 ± 0.72	2.98 ± 0.70	< 0.001
Sex			< 0.001
Male	7374 (53.89%)	2377 (58.50%)	
Female	6309 (46.11%)	1686 (41.50%)	
Smoking status			0.039
Current smoker	947 (6.92%)	233 (5.73%)	
Ever smoker	111 (0.81%)	28 (0.69%)	
Never	2517 (18.40%)	737 (18.14%)	
Drinking status			0.216
Current drinker	116 (0.85%)	36 (0.89%)	
Ever drinker	352 (2.57%)	102 (2.51%)	
Never	3107 (22.71%)	860 (21.17%)	
Family history of diabetes			0.098
No	13,584 (99.28%)	4023 (99.02%)	
Yes	99 (0.72%)	40 (0.98%)	
Follow-up (year)	3.07 ± 0.89	3.34 ± 0.91	< 0.001

Continuous variables were summarized as mean (SD) or medians (quartile interval); categorical variables were displayed as percentage (%).

*BMI* body mass index, *SBP* systolic blood pressure, *DBP* diastolic blood pressure, *TG* triglyceride, *TC* total cholesterol, *HDL-c* high-density lipoprotein cholesterol, *LDL-c* low-density lipoprotein cholesterol, *AST* aspartate aminotransferase, *ALT* alanine aminotransferase, *BUN* blood urea nitrogen, *Scr* serum creatinine, *FBG* fasting plasma glucose, *TyG index* triglyceride glucose index.

### Incidence of IFG in participants

**Figure 2:**
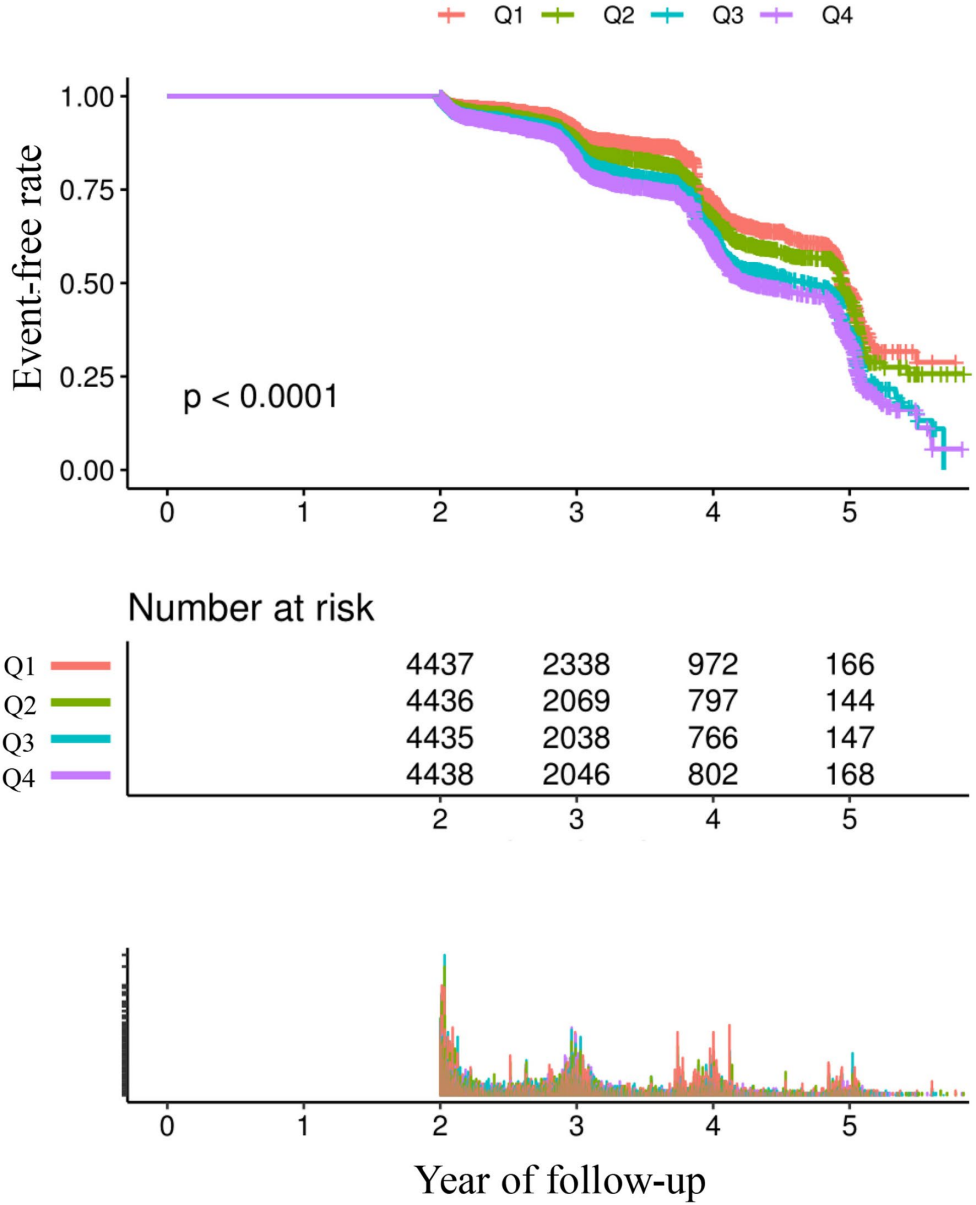
The Kaplan–Meier curve depicts the probability of IFG occurrence, stratified by the TyG index. The graph demonstrates that the likelihood of IFG occurrence gradually increases with higher TyG index values. This suggests that patients with the highest TyG index have the highest probability of experiencing IFG.

Table 2 and Fig. 2 describe the incidence rates of IFG. Among the participants, 4063 (22.9%) developed IFG. Participants were divided into subgroups based on the quartiles of the TyG index. The incidence rates of IFG per 1000 person-years were 58.89, 64.26, 79.69, and 90.33 for each TyG index quartile. The incidence rates of IFG in each TyG index quartile were as follows: Q1: 18.89%, Q2: 19.93%, Q3: 24.60%, and Q4: 28.08%. Participants with the highest TyG index (Q4) had a higher risk of developing IFG compared to those with the lowest TyG index (Q1) (trend P < 0.001).

**Table 2:** The incidence rate of IFG (% or per 1000 person-year).

TyG index (quartile)	Participants (n)	IFG (n)	Incidence (%) (95% CI)	Per 1000 person-year
Total	17,746	4063	22.90 (22.28–23.51)	73.19
Q1	4437	842	18.98 (17.82–20.13)	58.99
Q2	4436	884	19.93 (18.75–21.10)	64.26
Q3	4435	1091	24.60 (23.33–25.87)	79.69
Q4	4438	1246	28.08 (26.75–29.40)	90.33
P for trend				< 0.001

### Multivariable analysis using Cox proportional hazards regression model

In Table 3, the association between the TyG index and the risk of IFG is examined using multivariable analysis with the Cox proportional hazards regression model. The results show that in the crude model, the hazard ratio for the TyG index was statistically significant. When adjusting for age and sex (Model I), the hazard ratio remained significant but slightly decreased. Further adjustments in Model II, which included various additional factors, still showed a significant association between the TyG index and the risk of IFG. Additionally, after adjustments, consistent trends were observed across all quartiles of the TyG index, further highlighting the significant association between the TyG index quartiles and the risk of IFG. These findings suggest that the TyG index is independently associated with an increased risk of IFG, even after accounting for potential confounding factors. This underscores the importance of considering the TyG index in assessing the risk of IFG in clinical and research settings.

**Table 3:** Association between TyG index and risk of IFG in different models.

Exposure	Crude model (HR, 95% CI) P	Model I (HR, 95% CI) P	Model II (HR, 95% CI) P	GAM (HR, 95% CI) P
TyG index	1.43 (1.35, 1.51) < 0.0001	1.44 (1.36, 1.52) < 0.0001	1.43 (1.27, 1.60) < 0.0001	1.48 (1.32, 1.66) < 0.0001
(Quartile)				
Q1	Ref	Ref	Ref	Ref
Q2	1.18 (1.08, 1.30) 0.0005	1.18 (1.08, 1.30) 0.0005	0.99 (0.85, 1.15) 0.8550	0.99 (0.85, 1.16) 0.9454
Q3	1.48 (1.35, 1.62) < 0.0001	1.49 (1.36, 1.63) < 0.0001	1.35 (1.16, 1.58) 0.0001	1.38 (1.18, 1.61) < 0.0001
Q4	1.65 (1.51, 1.80) < 0.0001	1.66 (1.52, 1.82) < 0.0001	1.62 (1.37, 1.90) < 0.0001	1.71 (1.45, 2.02) < 0.0001
P for trend	< 0.0001	< 0.0001	< 0.0001	< 0.0001

Crude model: we did not adjust other covariates.

Model I: we adjusted age, sex.

Model II: we adjusted age, sex, SBP, DBP, BMI, ALT, AST, BUN, Scr, TC, LDL-C, HDL-c, family history of diabetes, drinking status, and smoking status.

Model III: we adjusted age(smooth), sex, SBP (smooth), DBP (smooth), BMI (smooth), BUN (smooth), Scr (smooth), ALT (smooth), AST (smooth), TC (smooth), LDL-C(smooth), HDL-c (smooth), smoking status, drinking status, family history of diabetes. *HR* hazard ratios, *CI* confidence, *Ref* reference.

### Sensitivity analysis

We conducted a series of sensitivity analyses to ensure the validity of our findings. Initially, in Model III using the generalized additive model (GAM) with additional smooth terms for various variables, we observed a HR of 1.48 (95% CI 1.32–1.66, P < 0.0001), indicating a significant association (Table 3). By excluding participants with a BMI ≥ 28 kg/m^2^ and adjusting for confounding factors, the results consistently showed a positive association between the TyG index and the risk of impaired fasting glucose (IFG) with an HR of 1.42 (95% CI 1.26–1.61, P < 0.0001). Moreover, we conducted sensitivity analyses by excluding individuals aged ≥ 80 years, and the association between the TyG index and IFG risk remained significant (risk ratio = 1.45, 95% CI 1.29–1.64, P < 0.0001) after adjustments. Additionally, when analyzing participant’s SBP < 140 mmHg, the risk ratio was 1.51 (95% CI 1.31–1.74, P < 0.0001) (Table 4). After considering all these sensitivity analyses, we can confidently conclude that our results are dependable and robust, emphasizing the consistent positive association between the TyG index and the risk of IFG.

**Table 4:** Association between TyG index and the risk of IFG in different sensitivity analyses.

Exposure	Model I (HR, 95% CI) P	Model II (HR, 95% CI) P	Model III (HR, 95% CI) P
TyG index	1.42 (1.26, 1.61) < 0.0001	1.45 (1.29, 1.64) < 0.0001	1.51 (1.31, 1.74) < 0.0001
(Quartile)			
Q1	Ref	Ref	Ref
Q2	0.96 (0.82, 1.13) 0.6550	1.05 (0.90, 1.24) 0.5228	1.05 (0.87, 1.27) 0.6229
Q3	1.32 (1.12, 1.55) 0.0010	1.40 (1.19, 1.65) < 0.0001	1.58 (1.30, 1.92) < 0.0001
Q4	1.58 (1.33, 1.88) < 0.0001	1.68 (1.41, 2.00) < 0.0001	1.91 (1.55, 2.35) < 0.0001
P for trend	< 0.0001	< 0.0001	< 0.0001

Crude model I was a sensitivity analysis performed after excluding participants with BMI ≥ 28 kg/m^2^. we adjusted age, sex, SBP, DBP, ALT, AST, BUN, Scr, TC, LDL-C, HDL-c, family history of diabetes, drinking status, and smoking status.

Model II was a sensitivity analysis performed after excluding participants with age ≥ 80 years old. We adjusted age, sex, SBP, DBP, BMI, ALT, AST, BUN, Scr, TC, LDL-C, HDL-c, family history of diabetes, drinking status, and smoking status.

Model III was a sensitivity analysis performed after excluding participants with SBP ≥ 140 mmHg. We adjusted age, sex, SBP, DBP, BMI, ALT, AST, BUN, Scr, TC, LDL-C, HDL-c, family history of diabetes, drinking status, and smoking status. *HR* hazard ratios, *CI* confidence, *Ref* reference.

### Association between the TyG index and the risk of IFG

**Figure 3:**
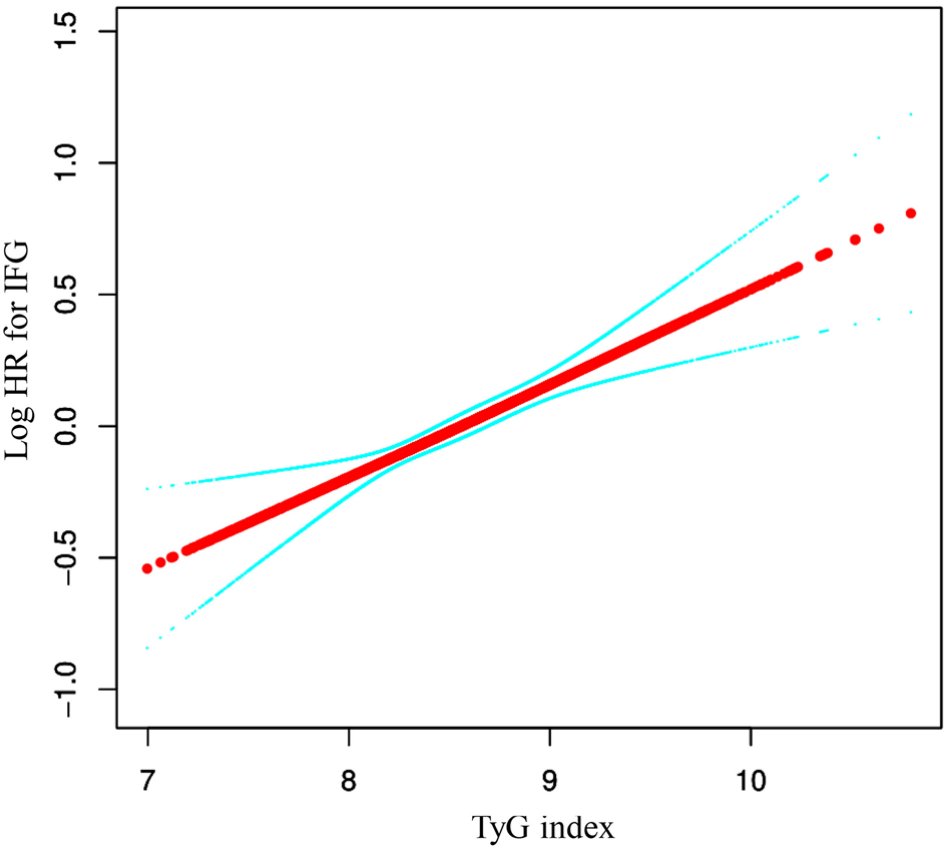
The association between TyG index and the risk of IFG is linear. A linear relationship between them was detected after adjusting for age, sex, SBP, DBP, BMI, ALT, AST, BUN, Scr, TC, LDL-C, HDL-c, family history of diabetes, drinking status, and smoking status.

In our study, we found a clear linear association between the TyG index and the risk of IFG. By utilizing a Cox proportional hazards regression model with cubic spline functions, we were able to evaluate this association and confirmed that it is indeed linear (Fig. 3). This finding suggests that as the TyG index increases, so does the risk of developing IFG.

### Subgroup analysis results

As illustrated in Table 5, a detailed subgroup analysis and interactions were conducted. Firstly, we stratified the analysis by age, gender, BMI, systolic and diastolic blood pressures, smoking and drinking habits, and a family history of diabetes. Through multivariate Cox proportional hazards models, we found statistically significant differences in HR (95% CI) between the 60–70 years old and 70–80 years old groups. However, there were no statistically significant differences in HR (95% CI) between the 80–90 years old and ≥ 90 years old groups. Additionally, in the subgroups of BMI < 18.5 kg/m^2^ and ≥ 28 kg/m^2^, there were no statistically significant differences in HR (95% CI). Secondly, we performed an interaction analysis for all subgroups, and we found that no significant interactions were observed between these variables and the TyG index (all interaction P > 0.05).

**Table 5:** Effect size of TyG index on IFG in prespecified and exploratory subgroups.

Variable	HR (95% CI)	P-value	P for interaction
Age, yeas			0.6378
60–70	1.44 (1.26, 1.64)	< 0.0001	
70–80	1.45 (1.16, 1.82)	0.0011	
80–90	1.13 (0.77, 1.64)	0.5336	
≥ 90	2.11 (0.14, 30.92)	0.5780	
BMI (kg/m^2^)			0.2039
< 18.5	0.79 (0.35, 1.75)	0.556	
≥ 18.5, < 25	1.53 (1.32, 1.77)	< 0.0001	
≥ 25, < 28	1.43 (1.19, 1.72)	0.0002	
≥ 28	1.20 (0.90, 1.60)	0.2047	
Sex			0.4588
Male	1.38 (1.20, 1.59)	< 0.0001	
Female	1.49 (1.27, 1.76)	< 0.0001	
SBP (mmHg)			0.0596
< 140	1.55 (1.36, 1.78)	< 0.0001	
≥ 140	1.28 (1.08, 1.52)	0.0047	
DBP (mmHg)			0.7253
< 90	1.43 (1.27, 1.62)	< 0.0001	
≥ 90	1.37 (1.10, 1.72)	0.0057	
Family history of diabetes			0.5347
Yes	1.43 (1.27, 1.60)	< 0.0001	
No	0.74 (0.09, 6.30)	0.7869	

Note 1: Above model adjusted for age, sex, SBP, DBP, BMI, ALT, AST, BUN, Scr, TC, LDL-C, HDL-c, family history of diabetes, drinking status, and smoking status.

Note 2: In each case, the model is not adjusted for the stratification variable when the stratification variable was a categorical variable.

## Discussion

This retrospective cohort study aims to investigate the association between the TyG index and the risk of IFG. We found that the risk of IFG increases with the TyG index, and a linear association between them was observed. The highest quartile of the TyG index has a 1.62 times higher risk of IFG compared to the lowest quartile. These results suggest that the TyG index may be an effective indicator for monitoring IFG.

Globally, the incidence of prediabetes is on the rise. According to estimates by the World Health Organization, in 2021, the global diabetes population was approximately 537 million. The prevalence of diabetes in China rose significantly from 22.5 million individuals in the year 2000 to a staggering 140.9 million by 2021^25^. The number of people with prediabetes exceeds these figures. A study in the United States suggests that about one-third of adults are considered to have prediabetes^26^. The prevalence of prediabetes in Chinese adults is alarmingly high, with an estimated rate of 50.1%. the prevalence of prediabetes is higher in men, with a rate of 52.1%, compared to women at 48.1%. This suggests that one out of every two Chinese adults may be at risk of developing diabetes if proper measures are not taken^27^. Studies have indicated that the standardized incidence rate of prediabetes in the overall population of China is 62.6 cases per 1000 person-years (73.8 cases per 1000 person-years among males and 51.2 cases per 1000 person-years among females)^28^. Additionally, another cohort study in China, which included 4093 Chinese adults with a median follow-up time of 3.25 years, found that 26.2% of participants developed prediabetes^29^. IFG is a state of prediabetes, and the primary outcome variable studied here was IFG. Our study reveals that over a 5-year period, 22.9% of participants developed impaired fasting glucose (IFG), with an incidence rate of 73.19 per 1000 person-years. The differences in prediabetes incidence rates among various studies could be attributed to variations in participants’ age, follow-up duration, and ethnicity. Notably, less than 11% of individuals are aware of their condition^30^. Therefore, identifying factors leading to IFG is crucial for preventing diabetes and its complications.

A cohort study in China, involving 201,298 individuals, found that a high TyG index was independently associated with the risk of developing diabetes (HR) = 3.34; 95% CI 3.11–3.60)^31^. Another cohort study involving 4543 Chinese adults utilized logistic regression analysis adjusted for several confounders, showing that for each standard deviation increase in the TyG index, the risk of prediabetes increased by 1.38 times (95% CI 1.28–1.48)^29^. Hence, we hypothesize that an increase in the TyG index might be related to an increased risk of IFG in the elderly. Unfortunately, reports on their relationship are scarce. Linhao Zhang et al. found a non-linear association between the TyG index and impaired fasting blood glucose by analyzing data from 25,159 patients^32^. Xiaoxia Li et al. demonstrated that, based on baseline data, logistic analysis showed that after multivariate adjustment, the TyG index was significantly positively correlated with IFG. However, the association was not significant after further adjustment (HR = 1.06; 95% CI 0.58–1.96; P for trend = 0.784)^33^. This study may differ from ours because the sample size was not as large as ours. Additionally, the average age of participants in the study by Li et al. (48.2 years) was much younger compared to our study participants (67.01 years). This difference in age may be a significant factor contributing to the discrepancies in study outcomes. However, our study found a positive, linear association between the TyG index and the risk of IFG in the elderly. Our research adds to the existing literature, supporting the hypothesis that an elevated TyG index is associated with an increased IFG risk. Compared to other studies, in our research, the TyG index was utilized in both categorical and continuous forms to examine the association between TyG index and the risk of IFG. Our aim was to minimize loss of information and accurately measure the association between the two variables. Furthermore, the confounding factors adjusted in our study differ from previous ones. We adjusted for a wider variety of variables, such as age, gender, systolic blood pressure, diastolic blood pressure, body mass index, alanine aminotransferase, aspartate aminotransferase, blood urea nitrogen, serum creatinine, total cholesterol, low-density lipoprotein cholesterol, high-density lipoprotein cholesterol, diabetes family history, alcohol consumption, and tobacco use. Sensitivity analysis confirmed that this association persists even after excluding participants with age ≥ 80 years, BMI ≥ 28 kg/m^2^, and SBP ≥ 140 mmHg. Additionally, subgroup analyses and interaction tests on age, DBP, BMI, SBP, gender, and family history of diabetes showed no interactions, confirming the stability of the association between the TyG index and IFG risk. This finding provides a reference for clinical interventions to reduce the probability of IFG in the elderly by targeting the TyG index.

The study has several strengths. Firstly, we have established a linear association between the TyG index and the risk of IFG among the elderly for the first time. In addition, the research involved a substantial group of 17,746 senior citizens and accounted for variables like BMI, age, TC, SBP, BUN, ALT, AST, DBP, Scr, LDL-C, HDL-C, gender, diabetic family history, alcohol consumption, and tobacco use to reduce possible distortions. To ensure the reliability and robustness of the results, sensitivity analysis was performed. Moreover, subgroup analysis and tests for interactions were conducted. The results indicate that the TyG index has different effects on IFG risk across various subgroups, further validating the experiment’s stability.

Despite these strengths, our study has several limitations. First, the average follow-up duration of the study participants was only 5.0 years, which is relatively short. Second, participants were only followed up once, with information available only at two time points (baseline and follow-up). Third, there were no available serum insulin level data, thus the predictive value of the TyG index could not be compared. Fourth, values for impaired glucose tolerance at 2 h or HbA1c were not collected, hence further analysis of the association between the TyG index and changes in glucose tolerance and HbA1c was not possible. Fifth, the study focused only on individuals aged over 60 years, and may not represent all populations; further research should expand to include various age groups. In addition, the original study did not collect data on uric acid. Therefore, we are unable to include uric acid in the current analysis. Future research should consider collecting and analyzing uric acid and other potentially influential factors on IFG to comprehensively elucidate their roles.

## Conclusion

This study revealed a linear association between the TyG index and the IFG in Chinese individuals aged over 60. Understanding this linear association can help clinicians identify high-risk elder individuals and implement focused interventions to reduce the risk of developing diabetes.

## Methods

### Study design

This study utilized data from a previous retrospective cohort study conducted by Chinese researchers^34^. The target independent variable was the TyG at baseline. The outcome variable was the development from normoglycemia to IFG at follow-up.

### Data source

Access to the original dataset was granted at no cost through the DATADRYAD platform (http://www.datadryad.org), courtesy of Ying Chen et al.^34^. In accordance with Dryad’s usage policy, the data is available for academic and research purposes, allowing users to share, adapt, alter, and build upon the material, provided it is not for commercial use and proper attribution is given to the original authors and source. The dataset was sourced from a publicly accessible study published in 2018 titled “Association of body mass index and age with diabetes onset in Chinese adults: a population-based cohort study,” which can be found at 10.1136/bmjopen-2018-021768. For those interested, the dataset can be retrieved from the following link: 10.5061/dryad.ft8750v.

### Ethics approval and consent to participate

The prior study received ethical approval from the Rich Healthcare Group Review Board. Given that the current study involves a secondary analysis of existing data, there was no need for obtaining informed consent or additional ethical approval. All methods were performed in accordance with the relevant guidelines and regulations.

### Research population

The primary research included 685,277 individuals aged 20 years and above, all of whom had undergone a minimum of two health assessments. The study focused on participants who, during follow-up, had FPG levels ranging from 6.1 to 6.9 mmol/L without any prior diagnosis of diabetes. The initial selection excluded participants based on several factors: (1) lack of detailed information regarding weight, height, or gender; (2) BMI values outside the normal range (< 15 kg/m^2^ or > 55 kg/m^2^); (3) intervals between visits shorter than 2 years; (4) missing FPG readings; (5) individuals diagnosed with diabetes at the start or with uncertain diabetes status at the time of follow-up. Following these criteria, the study retained 211,833 participants.

**Figure 4:**
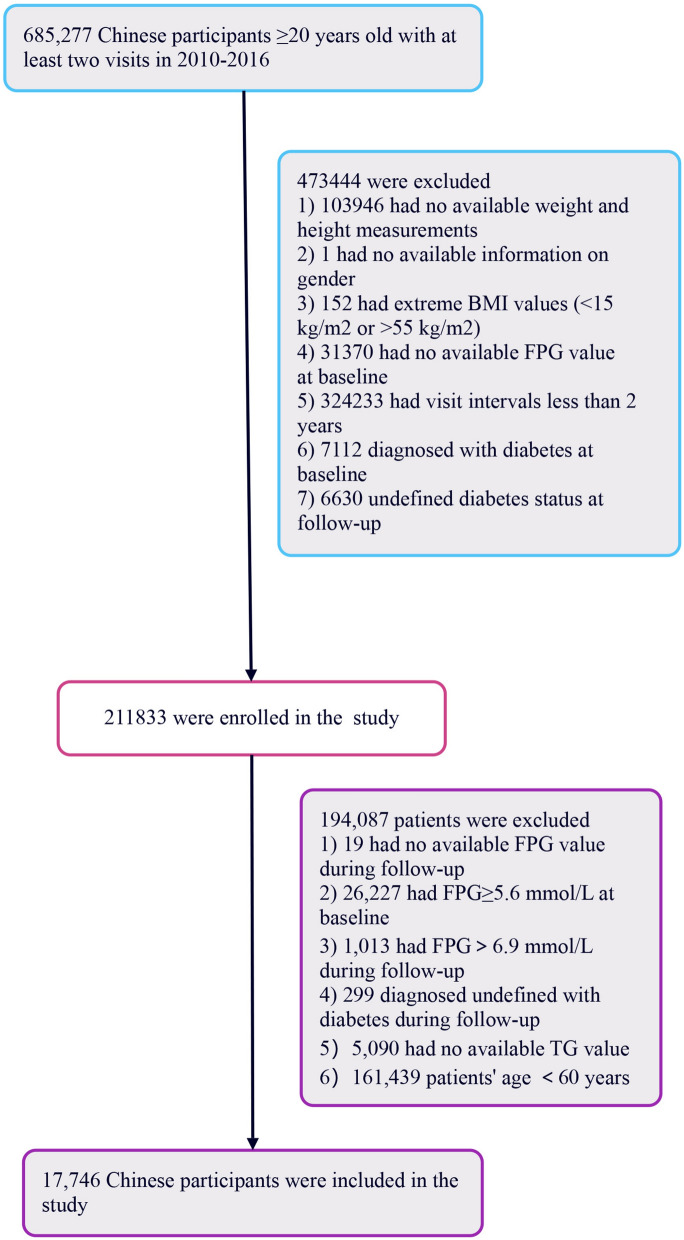
Flowchart illustrating the selection process of study participants.

Further analysis led to the exclusion of an additional 194,087 participants for reasons including: (1) absence of follow-up FPG readings, (2) baseline FPG levels ≥ 5.6 mmol/L, (3) FPG levels > 6.9 mmol/L during follow-up, (4) unclear diabetes diagnosis at follow-up, and (5) lack of triglyceride (TG) values or being less than 60 years of age. Elder people are defined as those aged over 60 years old or older^25^. Ultimately, the study included 17,746 participants. The process of selecting participants for this study is depicted in Fig. 4.

### Data collection

In this study, data collection included demographic information such as age, diastolic blood pressure (DBP), systolic blood pressure (SBP), height, and weight, from which body mass index (BMI) was calculated. To ensure consistency in data collection, staff received specialized training focusing on demographic data and key measurements, including blood pressure. Tests were uniformly conducted in a standardized laboratory environment for triglycerides (TG), high-density lipoprotein cholesterol (HDL-c), total cholesterol (TC), blood urea nitrogen (BUN), serum creatinine (Scr), alanine aminotransferase (ALT), low-density lipoprotein cholesterol (LDL-c), and FPG. Additionally, the study collected information on the patients’ smoking and drinking histories, defining current drinking as 1, former drinking as 2, never drinking as 3, and unknown drinking status as 4. Similarly, current smoking was coded as 1, former smoking as 2, never smoking as 3, and unknown smoking status as 4. A family history of diabetes mellitus is obtained by inquiring whether the patient’s first-degree relatives (parents, grandparents, siblings, etc.) have diabetes.

### Outcome and definitions

At follow-up, our focus was on identifying individuals who had an impaired fasting glucose (IFG) condition. This was determined by having fasting plasma glucose (FPG) levels fall within the range of 5.6–6.9 mmol/L, with no reported cases of new-onset diabetes^35^. We defined “elderly patients” as aged over 60^36^.

### Missing data processing

In this study, the number of participants with missing data was as follows: 7 (0.00%) each for DBP and SBP, 1 person (0.00%) for TC, 869 (0.69%) for ALT, 72640 (58%) for AST, 60634 (48.39%) for LDL-c, 6192 (4.9%) for Scr, 60579 (48.34%) for HDL-c, and 11452 (9.1%) for BUN. To mitigate the uncertainty caused by missing data, this study utilized multiple imputation techniques. The imputation model included ALT, LDL-c, AST, Scr, HDL-c, and BUN, with 5 iterations using linear regression. Data analysis assumed that missingness was random (MAR)^37,38^.

### Statistical analysis

The main variable examined in this research is the TyG index, which is defined by the equation: TyG index = ln [FPG (mg/dL) × TG (mg/dL)/2]^39–41^. We divided it into four quartiles and considered it as a continuous variable. We presented the mean and standard deviation for continuous variables that followed a normal distribution, while the median was reported for data that did not follow a normal distribution. For categorical variables, we presented the frequency and proportion of the data in our study. To analyze the differences between different TyG index groups, we utilized the Kruskal–Wallis *H* test for data that was not normally distributed, one-way analysis of variance for normally distributed data, and the chi-square test for categorical variables.

We developed numerous models to evaluate the association between the TyG index and the risk of IFG: a baseline model without any adjustments, a simplified model adjusting for gender and age only (Model I), and a comprehensive model adjusting for multiple covariates (Model II: recording a variety of demographic and health-related variables, such as age, gender, BMI, blood pressure, liver enzymes, cholesterol levels, serum creatinine, family history of diabetes, alcohol consumption, and smoking status, in our study. From each model, we noted the effect size (hazard ratio HR) along with its 95% confidence interval (CI).

To validate our findings, we conducted a series of sensitivity analyses. By incorporating continuous variables into a generalized additive model (GAM) in curve form, we further confirmed the robustness of the results. Additionally, we conducted analyses using stratified Cox proportional hazards models in different subgroups (such as age, gender, blood pressure, smoking, and drinking status). Finally, we used likelihood ratio tests to examine whether there were interactions in the model, both in models including interaction terms and those without. All analyses were performed using Empower Stats (X&Y Solutions, Inc., Boston, MA, http://www.empowerstats.com), with a statistical significance level set at a two-sided P value less than 0.05.

### Ethics statement

The studies involving human participants were reviewed and approved by Rich Healthcare Group Review Board. The ethics committee waived the requirement of written informed consent for participation.

## Data Availability

The dataset was sourced from a publicly accessible study published in 2018 titled “Association of body mass index and age with diabetes onset in Chinese adults: a population-based cohort study,” which can be found at 10.1136/bmjopen-2018-021768. For those interested, the dataset can be retrieved from the following link: 10.5061/dryad.ft8750v.
